# Could additional operational fund improve the performance of health centres' underfunded public health programmes? Learning from the Sleman district experience

**DOI:** 10.1186/1471-2458-14-S1-O16

**Published:** 2014-01-29

**Authors:** Novita Krisnaeni

**Affiliations:** 1 Dinas Kesehatan Sleman, Yogyakarta 55511, Indonesia

## Background

Preventive and promotive programmes in government health centres are commonly underfunded. Aside from that, the central government allocates money based on vertical programmes category. To put public health programs as a high priority, the government decided to make an additional special allocation so that government health centres may improve their performance in the outcomes of underfunded programmes. This study documented the practice of using the implementing operational funds in all health centres of Sleman district in the 2012. In particular, we wanted to study the administration procedures in spending the funds and how they can achieve better programme coverage and better programme implementation. Understanding the appropriate use of the funds will be an important consideration for continuing the same policy in the future.

## Materials and methods

We used data from official periodical reports of the use of operational funds that were submitted to Sleman district health office. In addition, we conducted in-depth interviews in health centre managers on the benefits and challenges in the use of these funds.

## Results

Each health centre gained US$75,000 a year. Although all health centres spent all 100% of the operational funds available to them, meaningful achievements from the programme were hard to report. These conditions were related to overall weakness in programme implementation and management. Planning failed to address priority issues and the need-service gaps.

## Conclusions

This study reports benefits and problems in using a separate additional operational fund to strengthen public health programmes. It is hard to conclude about the effectiveness of the funds while the overall implementation of preventive and promotive programmes in health centres is weak. Alternative mechanisms need to be sought in order that the use of funds would improve the overall programme implementation.

